# Whole Exome-Sequencing of Pooled Genomic DNA Samples to Detect Quantitative Trait Loci in Esotropia and Exotropia of Strabismus in Japanese

**DOI:** 10.3390/life12010041

**Published:** 2021-12-27

**Authors:** Jingjing Zhang, Toshihiko Matsuo, Ichiro Hamasaki, Kazuhiro Sato

**Affiliations:** 1 Regenerative and Reconstructive Medicine (Ophthalmology), Graduate School of Interdisciplinary Science and Engineering in Health Systems, Okayama University, Okayama 700-8558, Japan; zhangjjgg@yahoo.co.jp; 2 Department of Ophthalmology, Okayama University Hospital, Okayama 700-8558, Japan; hamasaki_16@yahoo.co.jp; 3 Barley and Wild Plant Resource Center, Institute of Plant Science and Resources (IPSR), Okayama University, Kurashiki 710-0046, Japan; kazsato@rib.okayama-u.ac.jp

**Keywords:** strabismus, esotropia, exotropia, diploid plant, human, quantitative trait locus (QTL), pooled genomic DNA, chromosome, single nucleotide polymorphism (SNP), whole exome sequencing

## Abstract

Background: Esotropia and exotropia are two major phenotypes of comitant strabismus. It remains controversial whether esotropia and exotropia would share common genetic backgrounds. In this study, we used a quantitative trait locus (QTL)-sequencing pipeline for diploid plants to screen for susceptibility loci of strabismus in whole exome sequencing of pooled genomic DNAs of individuals. Methods: Pooled genomic DNA (2.5 ng each) of 20 individuals in three groups, Japanese patients with esotropia and exotropia, and normal members in the families, was sequenced twice after exome capture, and the first and second sets of data in each group were combined to increase the read depth. The SNP index, as the ratio of variant genotype reads to all reads, and Δ(SNP index) values, as the difference of SNP index between two groups, were calculated by sliding window analysis with a 4 Mb window size and 10 kb slide size. The rows of 200 “N”s were inserted as a putative 200-b spacer between every adjoining locus to depict Δ(SNP index) plots on each chromosome. SNP positions with depth < 20 as well as SNP positions with SNP index of <0.3 were excluded. Results: After the exclusion of SNPs, 12,242 SNPs in esotropia/normal group and 12,108 SNPs in exotropia/normal group remained. The patterns of the Δ(SNP index) plots on each chromosome appeared different between esotropia/normal group and exotropia/normal group. When the consecutive groups of SNPs on each chromosome were set at three patterns: SNPs in each cytogenetic band, 50 consecutive sliding SNPs, and SNPs in 4 Mb window size with 10 kb slide size, *p* values (Wilcoxon signed rank test) and Q values (false discovery rate) in a few loci as Manhattan plots showed significant differences in comparison between the Δ(SNP index) in the esotropia/normal group and exotropia/normal group. Conclusions: The pooled DNA sequencing and QTL mapping approach for plants could provide overview of genetic background on each chromosome and would suggest different genetic backgrounds for two major phenotypes of comitant strabismus, esotropia and exotropia.

## 1. Introduction

Comitant strabismus is the misalignment of two eyes that variously interferes with binocular vision and is mainly classified into esotropia and exotropia. The misalignment of two eyes in comitant strabismus remains constant in all directions of the gaze, which is a diagnostic hallmark to differentiate from paralytic (noncomitant) strabismus. Genetic background for comitant strabismus is indicated by twin studies and medical history analyses [1,2]. Environmental factors in pregnancy and delivery would also play a role in the development of comitant strabismus [2,3]. These facts suggest that comitant strabismus would be related to quantitative trait loci (QTLs), which have measurable phenotypic variations owing to genetic and environmental influences [4]. Many QTLs, related to human complex diseases, have been identified by a counterpart in the mammalian species whereas no study until now has focused on comitant strabismus [5]. The previous studies proposed some potential chromosomal loci for comitant strabismus by different groups of researchers but no agreement has been reached on the specific loci [6,7,8,9].

Bulk segregant analysis (BSA), using next generation sequencing, has been applied to a genomic DNA pooling method for QTL analysis [10]. Sequencing genomic DNA from pools of individuals (pool-sequencing) is a cost-effective alternative approach, which is designed to simplify the data-analyzing processes of sequencing genomic DNA of individual samples [11]. In plants or animals, artificial cross-breed makes pool-sequencing be a primary method to detect a gene/QTL [12,13]. In humans, however, the heterogeneity of independent individuals makes it harder to obtain distinct results from population-based pool-sequencing [14,15]. The previous studies have proposed methods to use whole-genome sequencing (WGS) or region hybridization capture of human DNA pools. However, in those studies, the diversity and complexity were underestimated in practical data, and data-processing performance was reduced by the large amount of data in the whole genome sequencing [16,17,18]. Under the circumstances, whole exome sequencing (WES) would be an efficient strategy to reduce the amount of data and to assess the protein-coding regions of the human genome, which would constitute about 85% of disease-causing DNA changes [19].

In this study, we used whole exome sequencing of pooled genomic DNAs of individuals to screen for susceptibility loci of strabismus by the method of QTL-sequencing pipeline, which has been developed for diploid plants. We aim to obtain a hint to whether esotropia and exotropia, as two major phenotypes of strabismus, might have different genetic backgrounds.

## 2. Materials and Methods

### 2.1. Subjects

Forty Japanese patients with concomitant strabismus (20 patients with esotropia and 20 with exotropia) and 20 normal individuals in the families were involved in this study. Gender information was as follows: 11 males and 8 females with one individual of unknown gender in esotropia group, 10 males and 10 females in exotropia group, and 6 males and 11 females with 3 individuals of unknown gender. The genomic DNA was extracted from peripheral leukocytes isolated from 5 mL blood in all individuals. The study conformed to the tenets of the Declaration of Helsinki and was approved by the Ethics Committee of Okayama University Graduate School of Medicine, Dentistry, and Pharmaceutical Sciences and Okayama University Hospital. According to the approved protocol, written consent was obtained from each participant. All methods were carried out in accordance with the relevant guidelines and regulations.

### 2.2. DNA Sequence

The DNA concentration was measured with a Qubit 2.0 fluorometer (Thermo Fisher Scientific, Waltham, MA, USA). Each genomic DNA sample was adjusted to the concentration of 5 ng/μL, and 20 samples (2.5 ng each) of individuals with esotropia, those with exotropia, and normal individuals were mixed into one sequencing library, separately [20]. Sample preparation and exome enrichment for next generation sequencing (NGS)-based locus mapping was performed by exome capture technique with Nextera Rapid Capture Exome Kit (8 rxn × 3 plex, Illumina, San Diego, CA, USA), and sequencing of the libraries was performed by multiplexing three libraries per lane with variant filter cutoff 30 on MiSeq according to the MiSeq System User’s Guide (Illumina, San Diego, CA, USA). The sequencing processes of the same pooled libraries were repeated, and then, the first and second sets of data in each group, esotropia, exotropia, and normal group, were combined to increase the read depth. The combined data for esotropia group and exotropia group were further combined to obtain the data for strabismus group, which was then compared with the normal group.

### 2.3. Sequencing Data Analysis and Generation of SNP Index

**Figure 1:**
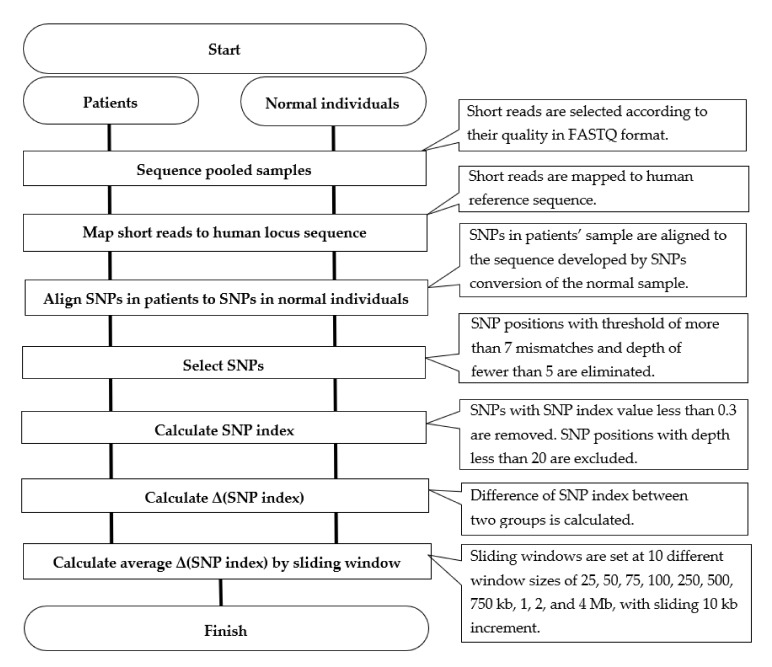
Flow chart of QTL-sequencing pipeline adapted for screening of human diseases, modified from the QTL-sequencing pipeline that was originally developed for diploid plants (Figure S5 in the reference [21]).

The QTL-sequencing pipeline [21,22] in diploid plants was modified and adapted to human diseases (Figure 1) and was used to analyze short reads from the libraries. A set of applications and scripts were installed on a local Linux server with 512 Gb main memory. Bulked reads were aligned to the human locus sequence (37.1 Mb), based on the GRCh38 human genome reference. The mismatch filter “Coval” was set to 6 [22,23]: SNP positions with the threshold of more than 7 mismatches and the depth of fewer than 5 were eliminated, as these SNPs were considered as sequencing or alignment errors. After mapping short reads to the reference sequence, the SNP index and Δ(SNP index) values were calculated for sliding window analysis. SNP index referred to the ratio of variant genotype reads to all reads, and Δ(SNP index) was the difference of SNP index between two groups [22]. The “window size” was configured in 10 different sizes as 25, 50, 75, 100, 250, 500, 750 kb, 1, 2, 4 Mb, and the “slide size” was set to 10 kb increment. To depict Δ(SNP index) plots on each chromosome, the rows of 200 “N”s were inserted as a putative 200-b spacer between the concatenated sequences of genes [22]. In statistical analysis, SNP positions with depth less than 20 were excluded. Furthermore, SNP positions with SNP index of <0.3 were excluded (Table 1).

## 3. Results

The two rounds of sequencing for each pool of 20 individuals generated 19,234,506 reads (2.8 Gb) for esotropia group, 19,886,144 reads (2.9 Gb) for exotropia group, and 16,324,940 reads (2.4 Gb) for normal group, respectively. The reads from each pool of 20 individuals for esotropia group, exotropia group, and normal group in two rounds of sequencing were tested for quality control (QC) in terms of duplicate level and mapping ability in the target regions, and all pools passed the QC test. In each pool, about 100,000 SNPs passed the filtering by Coval call such as low depth and strand bias (Table 1).

**Figure 2:**
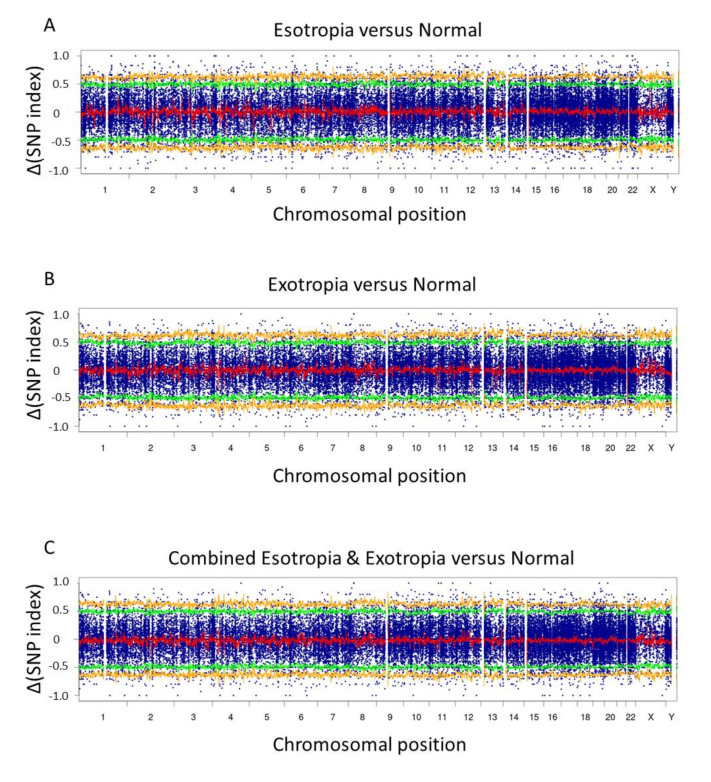
Δ(SNP index) plots on whole chromosomes in esotropia versus normal (**A**), exotropia versus normal (**B**), and combined esotropia and exotropia versus normal (**C**). The horizontal axis shows chromosomal position with the loci of genes concatenated with intervals of 200-b “N” as a spacer. The Δ(SNP index) was obtained by subtracting normal individuals’ SNP index from esotropic or exotropic or combined phenotypic patients’ SNP index. The thick red line represents the average of the ΔSNP index in each sliding window (window size, 4 Mb; slide size, 10 kb). Statistical confidence intervals under the null hypothesis of no quantitative trait loci are indicated by yellow (*p* < 0.01) and green (*p* < 0.05) lines. Positive and negative values in the ΔSNP index indicate higher and lower incidence, respectively, in esotropic or exotropic or combined phenotypic patients, compared with normal individuals. Note that the average of the ΔSNP index in each sliding window, as depicted by the thick red line, shows different patterns between the esotropia versus normal (**A**) and the exotropia versus normal (**B**), suggesting different genetic backgrounds in two phenotypes of strabismus, esotropia and exotropia.

Figure 2 shows the overall Δ(SNP index) distribution with the trendline and dense plots on the whole chromosomes in the best window size of 4 Mb with the 10 kb slide size. In comparison between esotropia group and normal group and also between exotropia group and normal group, small peaks were visualized along the Δ(SNP index) curve with the trendline and dense plots (Figure 2, Supplementary Figure S1) on some chromosomes. The patterns of the Δ(SNP index) curve on each chromosome in comparison between esotropia group and normal group appeared to be different from the patterns in comparison between exotropia group and normal group. When strabismus group was formed by combining esotropia group and exotropia group and was compared with the normal group, the patterns of the Δ(SNP index) curve on each chromosome appeared to be changed.

After SNP with a depth less than 20 was excluded and SNP index was limited to ≥0.3, the number of SNPs in esotropia/normal group was 12,242 in total and the number of SNPs in exotropia/normal was 12,108 in total (Table 1). The average depth of the SNPs was 39.3 ± 16.3 in esotropia/normal group and 39.1 ± 16.4 in exotropia/normal group as a mean ± standard deviation.

To statistically compare the Δ(SNP index) values between the esotropia/normal group and the exotropia/normal group, three regions for consecutive SNPs were defined: cytogenetic band region (cytoband), 50 consecutive sliding SNPs region (one SNP with the 25 preceding SNPs and the 24 following SNPs), and 4 Mb window with 10 kb sliding window (4 M 10 K window). After SNPs with a depth less than 20 were excluded, the number of SNPs in each cytoband was 14.7 ± 27.3 in esotropia/normal group and 14.4 ± 26.7 in exotropia/normal group as a mean ± standard deviation. The number of SNPs in each 50 SNPs region was 39.4 ± 4.4 in esotropia/normal group and 38.6 ± 4.7 in exotropia/normal group since the number of SNPs could not reach 50 toward the end of each chromosome. The number of SNPs in each 4 M 10 K window was 16.0 ± 27.9 in esotropia/normal group and 15.7 ± 27.5 in exotropia/normal group. Statistical comparison in each region was made by *p* values in Wilcoxon signed rank test with Bonferroni correction for multiple comparison to set a significant level and also by Q values in false discovery rate (FDR) with Storey correction to set a significant level at <0.05.

**Figure 3:**
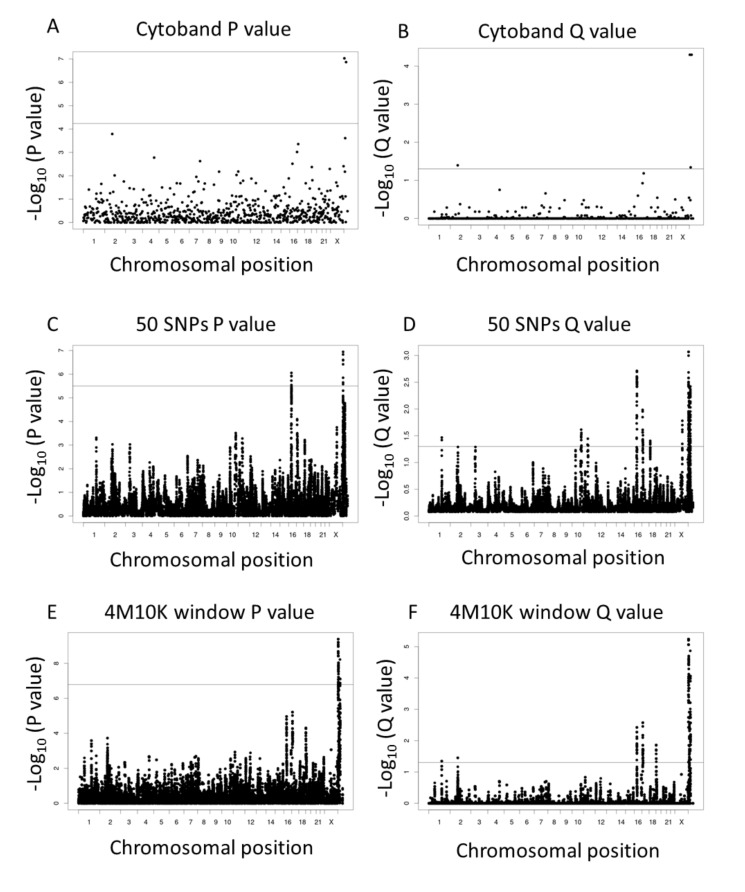
Manhattan plots of *p* values and Q values in statistical comparison between Δ(SNP index) in the esotropia versus normal (Figure 2A) and in the exotropia versus normal (Figure 2B). The consecutive groups of SNPs on each chromosome are set at three patterns: SNPs in each cytogenetic band (Cytoband, (**A**,**B**)), 50 consecutive sliding SNPs (50 SNPs, (**C**,**D**)), and 4 Mb window with 10 kb slide size (4 M 10 K, (**E**,**F**)). Horizontal lines show chromosomal positions and vertical lines show *p* values of Wilcoxon signed rank test with a significant line set by Bonferroni correction for multiple comparison (**left column**) and Q values of false discovery rate (FDR) with Storey correction to set a significant level at <0.05 (**right column**). For instance, *p* values and Q values on chromosome 16 are significant in 50 consecutive sliding SNPs (**C**,**D**), suggesting different genetic backgrounds on chromosome 16 in two phenotypes of strabismus, esotropia and exotropia. *p* values and Q values on Y chromosome would not be reliable due to the difference in male–female gender ratios.

*p* values and Q values are plotted as Manhattan plots along chromosomal positions in Figure 3. The distribution of *p* values and Q values along the chromosomal position showed similar patterns among three different methods for SNP comparison. A few chromosomal loci showed significant *p* values and Q values in comparison of Δ(SNP index) values between the esotropia/normal group and the exotropia/normal group. For instance, *p* values and Q values on chromosome 16 were significant in 50 consecutive sliding SNPs (Figure 3C,D), suggesting different genetic backgrounds on chromosome 16 in two phenotypes of strabismus, esotropia and exotropia. *p* values and Q values on Y chromosome would not be reliable due to the difference in male–female gender ratios.

## 4. Discussion

The previous pooled DNA methods for the human were based on single nucleotide polymorphisms (SNP) and corresponding allele frequency (AF) [11,14,17,24]. Genome-wide association study (GWAS) usually obtains gene regions or QTL by a Manhattan plot based on the allele frequency of a target SNP and its linkage disequilibrium (LD) SNPs. There has thus far been no study that targets a region in the pooled DNA-sequencing method. In this study, we attempted to use the pooled DNA-sequencing and QTL identification method developed for diploid plants to analyze the human genomic DNA pools.

In diploid inbreeding plants such as barley and rice, which are homozygotes with different phenotypes, SNP index in the genotypes would deviate from the baseline of 0.5 in target QTL and would be up or down to 1 or 0 under artificial selection by the human race. In contrast, the detection of susceptibility loci in human complex genetic diseases is more laborious compared with simple Mendelian disorders since the complex diseases are also influenced by other environmental factors. This fact makes the method for plants hard to be generalized to humans. However, since the genotype for the quantitative trait must reach a threshold that can be expressed [4], slight increase in a certain genotype might suggest a target locus. Under the circumstances, the difference in SNP index between the disease and the normal would reduce the fluctuation of the baseline to visualize SNPs in a robust way. In addition, the phenotype of a human complex disease would probably be the combination of multiple phenotypes, and thus, the listing of complete information in a single figure is essential to have overview on the genotypes.

The present results did not show a distinct peak of the Δ(SNP index) curve on each chromosome. The reasons are as follows. First, the SNP index plots in the target region in the individuals with binary segregation of strabismus and normal would not be around 0.5 as in the plants by crossing from inbred cultivars. Second, there is no distinct definition for the degree of strabismus as a quantitative character. The individuals with strabismus and normal individuals are located in the continuous range of phenotypic distribution and thus, would not have enough difference in the genotypes. Third, each QTL in human complex genetic diseases would make only small contributions to the phenotype. When many QTLs would affect a genetic trait in a multifactorial disease, the low effectiveness of each SNP would make indistinct the difference in the SNP index between two groups. Under the circumstances, a small peak might suggest a susceptible region, and the present results could provide a guide for identifying the strabismus susceptibility gene.

It remains unknown whether esotropia and exotropia would share common genetic backgrounds. When the data of esotropia group and exotropia group were combined to make the data of strabismus group, the Δ(SNP index) curves between strabismus group and normal group showed changes in peak patterns, in contrast with separate comparison of the normal group by esotropia group or exotropia group. There might be a statistical reason that the changes in the combined strabismus group might be caused by denser plots to fit curves. The different regions with peaks suggest that esotropia and exotropia might have different genetic backgrounds. As the other side of the coin, additional peaks in the Δ(SNP index) curves in esotropia group and exotropia group might suggest the regions that would give rise to diverse phenotypes of esotropia and exotropia. Technically speaking in general, the depth in the combined strabismus group would be saturated and doubling of the sample size would not provide additional information.

Pooled genomic DNA methods have been used for QTL-sequencing in plants and also for strain selection in animals [21,25]. In contrast with plants and animals, humans are definitely not homozygotes and separation of offspring cannot be obtained by crossing or natural inbreeding. Therefore, humans have more extensive allelic heterogeneity and highly diverse polymorphisms in pooled genomic DNA sequencing [11,16]. In the present study, for the first time and to the best of our knowledge, the pooled approach of sequence assembly and SNPs calling for the plant was applied to the human. The pooled method does focus on the universality in each group, which is derived from the proportion of shared SNPs among individuals but does not focus on the individuality that is generated by the diversity among individuals. In addition, the effect of polymorphisms might appear in opposite direction between different amounts of variants in a region [22], as known for the effect of oncogenes versus tumor suppressor genes. Therefore, the detection of variants in a region, rather than a single allele, is meaningful. In this sense, the pooled approach for the plant could be applied to the detection of susceptibility loci for a human disease.

In a pool of genomic DNAs, all individuals should share the same rare variants that would reach the significant allele frequency (not below 0.01) for filter and would not be omitted as noise. Thus, the previous study did not recommend the use of pool-sequencing methods in humans, based on the calculation of allele frequency [11]. In contrast, the other study suggested that the detection of human disease susceptibility would become meaningful only when pooling of a large amount of samples was obtained as shown in allele frequency of the GWAS approach [16]. The allele frequency is not an essential element in the pooled genomic DNA approach. The allele frequency is population-based, and the large sample size is required only for statistical power of case-control studies or case-parent studies [26]. Because the individuals in a pool are derived from the same population, the significance has been tested at the step of variant call.

The pooling of DNA creates new problems and challenges for accurate variant call and allele proportion estimation. The critical point is to ensure that all individuals in the pool would share the same weight as they would contribute equimolar of DNA to the pool [16]. Although twenty samples are equivalently mixed to a library after adjusted to the same concentration, the balance of variants for individuals cannot be confirmed because of the depth and randomness of the sequencing. In the present study, multiple window sizes, multiple “Coval” values, and multiple depth values were set to make figures repeatedly (10 × 6 × 5 = 300 times), and the most distinguished figure was selected (Figure 2). The average curve as the trendline was created to compensate for the instability of scatter plots, and the dispersion of natural SNP frequency was enhanced by the insertion of a spacer among loci to make the target region clearer [22]. In addition, we independently sequenced twice the pooled genomic DNA, and combined the reads in separate sequencing to reduce the instrumental errors.

The alleles that were present at low frequency in the pools would be possibly ignored as sequencing errors or noise in the variant call. The previous studies found that the allele frequency as a whole, for instance, the combination of several QTLs, was robust and reliable even when some rare variants were omitted [11,24]. In addition, the other study found that variant detection programs gave high balanced accuracy for datasets with varying per-sample depth of coverage and the number of samples per pool [27]. The accuracy will decrease since the depth of coverage decreases when the number of samples per pool increases. To identify rare variants and to avoid the confounding effect by sequencing errors, we selected twenty samples for a pool and sequenced twice the same pooled genomic DNA.

There are several limitations as follows. First, genetic analysis in a pooled manner missed the information of the haplotype. The haplotype phase is important since disease susceptibility might be associated with several low-frequency variants in coordination [28]. Although the detail of haplotypes could not be determined in the pooled method, we could detect a cluster of high Δ(SNP index) that would be located in the suspicious regions. In addition, the peak could be identified irrespective of the haplotype structure. Second, several alleles with a peak that are not at their expected frequencies might be due to linkage disequilibrium rather than are associated with the disease. Third, covariates can be adjusted corresponding to the phenotyping of individuals in GWAS whereas covariates cannot be adjusted in the pooled method [29]. Strabismus was not affected by covariates such as sex, age, and treatment [9]. Fourth, the genomic samples in one hospital might have a certain population structure that would give rise to false-positive variants [30]. The effect of the population structure would be rather eliminated by fitting a curve to show the difference in SNP index between strabismic patients and normal individuals from the same population. Fifth, the assembly reference of the plant is the homozygous parent, whereas the reference in humans is from the public database: the GRCh38 human genome reference. The reference in humans provides good approximation of the genomic DNA of any single individual [31].

Even with these limitations, we demonstrated in this study that SNP loci in chromosome 16, for instance, had significant difference in Δ(SNP index) between the esotropia/normal group and the exotropia/normal group. Chromosome 16 was shown to carry strabismus susceptibility loci detected by linkage analysis in Japanese [32] and Arab [33]. In addition, our previous linkage study, involving Japanese patients with esotropia and exotropia, suggested that esotropia and exotropia might have different susceptibility loci while both phenotypes of strabismus shared common susceptibility loci [9]. The QTL-sequencing pipeline for diploid plants [21,22] could be adapted to the human, and the Δ(SNP index) would be used as a key for detecting disease susceptibility loci in bird’s eye view on the whole chromosomes. Statistical comparison between the Δ(SNP index) would be further refined to reach a reliable conclusion by a new method in the future as for how the groups of SNPs in each comparison would be defined. The present methods in the QTL-sequencing pipeline for the human are unique in two points: (1) the number of patients and the redundancy of reads in a sample of pooled genomic DNA would be easily changed according to the scale of analysis, and (2) whole exome sequencing could allow the detection of de novo SNPs in the specific comparison of patients with the normal counterpart.

## 5. Conclusions

The pool-sequencing approach for the plant could provide an overview on possible different genetic backgrounds for esotropia and exotropia as two major phenotypes of comitant strabismus in humans and would give insight into susceptibility genes for comitant strabismus.

## Figures and Tables

**Table 1:** The number of SNPs at each step of exclusion.

Pairs	Esotropia (ET)/Normal (N)			Exotropia (XT)/Normal (N)			ET + XT/N		
	ET	N	ET/N	XT	N	XT/N	ET+XT	N	ET + XT/N
The number of SNP call co6cov3 (co = 6, depth ≥ 3)	83,732	83,732	83,732	83,107	83,107	83,107	98,730	98,730	98,730
SNPs with depth ≥ 20	16,383	15,897	12,717	15,905	15,678	12,473	33,204	13,851	13,711
SNP index ≥ 0.3	15,946	15,514	12,242	15,576	15,365	12,108	32,108	13,707	13,367

## Data Availability

The original data sheet, created and analyzed in the current study, is available from the corresponding author on reasonable request. The whole exome-sequencing data of the esotropia group, exotropia group, and normal group were submitted to the DNA Data Bank of Japan (DDBJ) and released on 16 July 2021 (accession number PRJDB11932).

## Supplementary Materials

The following is available online at www.mdpi.com/article/10.3390/life12010041/s1, Figure S1: Δ(SNP-index) plots on each chromosome in esotropia versus normal (top), exotropia versus normal (middle), and combined esotropia and exotropia versus normal (bottom). The horizontal axis shows chromosomal position with the loci of genes concatenated with intervals of 200-b “N” as a spacer. The Δ(SNP index) was obtained by subtracting normal individuals’ SNP index from esotropic or exotropic or combined phenotypic patients’ SNP index. The thick red line represents the average of the ΔSNP index in each sliding window (window size, 4 Mb; slide size, 10 kb). Statistical confidence intervals under the null hypothesis of no quantitative trait loci are indicated by yellow (*p* < 0.01) and green (*p* < 0.05) lines. Positive and negative values in the ΔSNP index indicate higher and lower incidence, respectively, in esotropic or exotropic or combined phenotypic patients, compared with normal individuals.
